# Overexpression of *Kcnmb2* in Dorsal CA1 of Offspring Mice Rescues Hippocampal Dysfunction Caused by a Methyl Donor-Rich Paternal Diet

**DOI:** 10.3389/fncel.2018.00360

**Published:** 2018-10-23

**Authors:** Ming Yu, Li Guo, Nan Li, Kristin S. Henzel, Huating Gu, Xiufang Ran, Wei Sun, Shuai Liu, Yingchang Lu, Dan Ehninger, Yu Zhou

**Affiliations:** ^1^Department of Physiology and Pathophysiology, School of Basic Medical Sciences, Qingdao University, Qingdao, China; ^2^Department of Physiology, Binzhou Medical University, Yantai, China; ^3^Molecular and Cellular Cognition Lab, German Center for Neurodegenerative Diseases, Bonn, Germany; ^4^Institute of Brain Sciences and Related Disorders, Qingdao University, Qingdao, China

**Keywords:** BK channels, *Kcnmb2*, hippocampus, memory, offspring, paternal diet, DNA methylation

## Abstract

BK channels are known regulators of neuronal excitability, synaptic plasticity, and memory. Our previous study showed that a paternal methyl donor-rich diet reduced the expression of *Kcnmb2*, which encodes BK channel subunit beta 2, and caused memory deficits in offspring mice. To explore the underlying cellular mechanisms, we investigated the intrinsic and synaptic properties of CA1 pyramidal neurons of the F1 offspring mice whose fathers were fed with either a methyl donor-rich diet (MD) or regular control diet (CD) for 6 weeks before mating. Whole-cell patch-clamp recordings of CA1 pyramidal neurons revealed a decrease in intrinsic excitability and reduced frequency of inhibitory post-synaptic currents in MD F1 mice compared to the CD F1 controls. AAV-based overexpression of *Kcnmb2* in dorsal CA1 ameliorated changes in neuronal excitability, synaptic transmission, and plasticity in MD F1 mice. Our findings thus indicate that a transient paternal exposure to a methyl donor-rich diet prior to mating alters *Kcnmb2*-sensitive hippocampal functions in offspring animals.

## Introduction

Large-conductance Ca^2+^- and voltage-activated K^+^ channels (also known as BK, Maxi-K, or Slo1) are widely expressed in mammalian central nervous systems (Sausbier et al., 2006; Contet et al., 2016). By providing negative feedback modulation to changes in membrane voltage and intracellular Ca^2+^ concentration, BK channels play significant roles in regulating a range of physiological processes, including action potential firing (Jin et al., 2000; Gu et al., 2007), neurotransmitter release (Hu et al., 2001; Faber and Sah, 2003; Raffaelli et al., 2004; Griguoli et al., 2016), and smooth muscle contraction (Ko et al., 2008; Hill et al., 2010; Krishnamoorthy-Natarajan and Koide, 2016). Evidence in recent years demonstrates that BK channels also take part in the regulation of learning and memory processes (Matthews and Disterhoft, 2009; Typlt et al., 2013; Springer et al., 2014). Moreover, alterations in the expression and function of BK channels have been linked to cognitive impairments (Wang F. et al., 2015; Wang L. et al., 2015; Contet et al., 2016).

BK channels are formed by a pore-forming α-subunit as well as tissue-specific β_1_–β_4_ auxiliary subunits, which are encoded by the *Kcnmb1–4* genes, respectively. Modulations by auxiliary subunits endow BK channels with diverse functions in different mammalian tissues and cell types (Orio et al., 2002; Wang et al., 2002; Li and Yan, 2016). Specifically, the β_2_ subunit, encoded by *Kcnmb2*, mediates rapid inactivation of the BK channel via its N-terminal ball and chain domain, thereby acting as one of the negative BK channel regulators (Wallner et al., 1999; Bentrop et al., 2001; Orio and Latorre, 2005; Lee and Cui, 2010).

In a previous study, we reported that transient exposure of male mice to a methyl donor-rich diet (MD) for 6 weeks before mating exerts intergenerational effects on cognitive functions in offspring animals (Ryan et al., 2017). Specifically, MD F1 offspring mice showed memory impairments in two hippocampus-dependent tasks (Morris water maze and contextual fear conditioning), as well as impaired long-term potentiation (LTP) and hippocampal theta oscillations. Gene expression analyses revealed reduced expression of *Kcnmb2* in MD F1 mice, which were associated with elevated *Kcnmb2* promoter methylation (Ryan et al., 2017). AAV-based overexpression of *Kcnmb2* in dorsal hippocampus improved spatial learning and memory in the Morris water maze in MD F1 animals, indicating that reduced *Kcnmb2* expression is linked to MD F1-associated learning and memory impairments.

It remains to be further clarified how reduced *Kcnmb2* expression leads to LTP and memory deficits in MD F1 offspring. Therefore, in this study, we assessed intrinsic excitability, synaptic transmission and plasticity in CA1 pyramidal neurons of both MD and CD F1 offspring mice. We also addressed if overexpression of *Kcnmb2* in the CA1 region of the dorsal hippocampus can rescue the neurophysiological alterations observed in MD F1 offspring mice.

## Materials and Methods

### Mice

For the experiments described here, we used the F1 offspring of C57BL/6J males that were transiently exposed to either the methyl donor-rich diet (MD, a specialized 3MS ZM diet) or the control diet (CD, a standard Teklad global 18% protein rodent-breeding diet) for a period of 6 weeks prior to mating them with 129S6/SvEv females, as previously described in detail (Ryan et al., 2017). Specifically, the 3MS ZM diet was supplemented with the following (per 1 kg chow): 7.5 g L-methionine, 15 g choline, 15 g betaine, 15 mg FA, 1.5 mg vitamin B12, and 150 mg zinc (Ryan et al., 2017). The mice were group-housed (two to five per cage) under a 12:12 h light/dark cycle and were given free access to water and food throughout the experiment. Adult CD and MD F1 offspring with matched age (4 to 8 months old when each experiment was conducted) were assessed in balanced sex ratios. All experiments were conducted blind to group assignment. The animal protocols used here were approved by the Chancellor’s Animal Research Committee at Qingdao University (in accordance with National Institutes of Health guidelines).

### Hippocampal Slice Preparations and Electrophysiological Recordings

#### Field EPSP Recordings

Mice were anesthetized using isoflurane and were rapidly decapitated. Hippocampal slices (400 μm) were sectioned in oxygenated ice-cold artificial cerebrospinal fluid (ACSF) containing (in mM): 120 NaCl, 1.25 NaH_2_PO_4_, 3.5 KCl, 1.3 MgCl_2_, 26 NaHCO_3_, 2.5 CaCl_2_, 10 D-glucose. Slices were recovered in a submerged chamber containing ACSF at room temperature for at least 1 h prior to recordings. During recordings, slices were continuously perfused with ACSF at a rate of ∼2 ml/min and at 32 ( ± 1°C).

Field excitatory post-synaptic potentials (fEPSPs) at Schaffer collateral-CA1 (SC-CA1) synapses were evoked every 30 s with FHC bipolar platinum stimulating electrodes as previously described (Cui et al., 2016). The input-output curve of synaptic transmission was generated by varying stimulus intensity from 10 to 100 μA and measuring the initial slope of the fEPSPs. Paired-pulse ratio (PPR) was determined by dividing the initial slope of the second fEPSP by that of the first (fEPSP2/fEPSP1) with different inter-stimulus intervals of 10, 25, 50, 100, 200, and 400 ms, respectively. LTP at SC-CA1 synapses was induced by a single tetanus of 100 pulses at 100 Hz (100 Hz, 1 s). All test stimuli and tetanus pulses were 100 μs in duration and 1/3–1/2 maximal stimulation strength (100 μA). All the field recording data were filtered at 1 kHz and digitized at 10 kHz. Data were acquired using Clampex 10 (Molecular Devices), and analyzed using Clampfit 10 (Axon Instruments). All chemicals used for electrophysiological recordings were purchased from Sigma.

#### Whole-Cell Recording

Hippocampal coronal slices (350 μm in thickness) were cut with a vibratome (VT-1000, Leica, Germany) in oxygenated (95% O_2_/5% CO_2_), ice-cold cutting solution (pH 7.4) containing (in mM) 30 Glucose, 2.5 KCl, 26 NaHCO_3_, 7 MgSO_4_, 1 NaH_2_PO_4_, 1CaCl_2_, 119 choline chloride, 1 kynurenic acid, 3 sodium pyruvate, and 1.3 sodium L-ascorbate. Slices were quickly transferred to the recovery solution containing (in mM) 85 NaCl, 2.5 KCl, 1.25 NaH_2_PO_4_, 0.5 CaCl_2_, 4 MgCl_2_, 24 NaHCO_3_, 25 glucose, and 50 sucrose to recover for 30 min at 36°C and then at least 1 h at room temperature before recording. Whole-cell patch-clamp recordings in voltage- or current-clamp mode were performed on CA1 pyramidal neurons in dorsal hippocampus analogous to previously described studies (Zhou et al., 2009; Springer et al., 2014).

CA1 neurons were identified both visually (with an upright microscope) and on the basis of firing properties (Fox and Ranck, 1981). In the context of current clamp recordings, patch electrodes (3–5 MΩ) were filled with internal solution (pH 7.30) which contained (in mM): 120 KMeSO_4_, 10 KCl, 10 Hepes, 0.2 EGTA, 0.3 Na_3_GTP, 4 Na_2_-ATP, 5 phosphocreatine, as well as 2 MgCl_2_. For voltage clamp recordings, patch pipettes (3–5 MΩ) were filled with a solution (pH 7.3) containing (in mM): 125 CsCl_2_, 5 NaCl, 4 Hepes, 0.2 EGTA, 0.2 NaGTP, 2 MgATP, 7 phosphocreatine, and 2 MgCl_2_. Both excitatory and inhibitory post-synaptic currents (EPSCs and IPSCs) were detected at a holding potential of -60 mV with 50 μM AP-5 and 50 μM picrotoxin (for EPSCs), or 3 mM kynuric acid (for IPSCs) present in the ACSF used for perfusion. Miniature inhibitory and excitatory post-synaptic currents (mIPSCs and mEPSCs) were recorded with the application of 1 μM TTX in external solutions. sEPSCs, mEPSCs, sIPSCs, and mIPSCs were analyzed using Mini Analysis Program. Event counts were carried out by experimenters blind to group identity.

To assess the intrinsic excitability of CA1 pyramidal neurons, a series of depolarizing currents (50 or 600 ms duration) stepping from -50 to 525 pA in 25 pA increments were injected through the patch electrode. Passive membrane properties of CA1 pyramidal neurons were examined as previously reported (Haghdoost-Yazdi et al., 2008; Springer et al., 2014). In brief, spike amplitude was measured from threshold to the peak of the first spike. Spike half-width was calculated as the first spike duration at half amplitude between the baseline and the peak of the first spike. The fast after-hyperpolarization potential (fAHP) amplitude was measured from spike threshold to the negative peak of the AHP within 4 ms from the time of the first spike peak. Data were acquired using digidata 1440A and pCLAMP 10.0 software with a sampling rate of 10 kHz. Only neurons that had sufficiently negative resting membrane potentials (≤-55 mV) without spontaneous firing were included in the analysis.

### Virus Microinjection Into CA1 of the Dorsal Hippocampus

Mice were anesthetized with isoflurane and placed in a stereotaxic frame. The skull was exposed and four holes were drilled above the CA1 region of dorsal hippocampus, according to the following coordinates: AP -1.8 mm, ML ± 1 mm, DV -1.4 mm from bregma and AP -2.5 mm, ML ± 2 mm, DV -1.7 mm from bregma. High titers (1.3 × 10^13^ GC/ml, Vector Biolabs) adeno-associated virus (AAV) engineered to overexpress *Kcnmb2* (AAV1-hSYN1-*mKcnmb2*-IRES-GFP-WPRE, AAV-Kcnmb2) or control virus (AAV1-hSYN1-GFP-WPRE, AAV-control) were stereotaxically injected into the dorsal CA1 region with Nanoliter 2010 (WPI) at a flow rate of 0.05 μl/min and a volume of 0.3 μl per injection site. After injection, the glass needle was left in place for an additional 10 min to ensure optimal virus diffusion. After surgery, mice were treated with antibiotics daily for 1 week and their health was monitored every day. The viral infection in the CA1 region was confirmed by GFP fluorescence. Relative expression of *Kcnmb2* in hippocampus was measured by real-time qRT-PCR. Electrophysiological experiments were performed 4–6 weeks following virus injection.

### RNA Extraction and qRT-PCR

Total RNA was extracted from the hippocampus with the PureLink^TM^ RNA Mini Kit (Thermo Fisher Scientific). RNA quantity and quality was determined using a NanoDrop 2000 Spectrophotometer (Thermo Fisher Scientific). Complementary DNA was synthesized from 1 μg of total RNA with SuperScript^TM^ III Reverse Transcriptase (Invitrogen). qPCR-based quantification of *Kcnmb2* was performed using a MasterCycler ep realplex PCR system (Eppendorf) and QuantiFast SYBR Green PCR Kit (Qiagen). The PCR cycling parameters were as follows: 95°C for 5 min, followed by 40 cycles of PCR reaction at 95°C for 5 s, 60°C for 30 s, 72°C for 30 s. *Actb* was used as housekeeping control for all samples. The expression of *Kcnmb2* in the MD F1 group was normalized to that observed in the CD F1 control group. The following PCR primer sequences were used: *Kcnmb2*-F *TGCAGGACCAACATCCTCTAAG*, *Kcnmb2*-R *CTTCAGAGCTGTCACAGTTTTCC*; *Actb*-F *CACTCTTCCAGCCTTCCTTC*, *Actb*-R *GTACAGGTCTTTGCGGATGT*.

### Immunofluorescence Staining

Mice were perfused transcardially with physiological saline solution followed by 4% paraformaldehyde (PFA). Brains were post-fixed in 4% PFA for 3 h and then transferred to 30% sucrose solution and stored at 4°C for 2 days. Hippocampal coronal section series (60 μm) were then collected. For immunofluorescence stainings, we used rabbit anti-KCNMB2 polyclonal antibody (1:800; Alomone labs) in conjunction with Alexa-568-conjugated goat anti-rabbit secondary antibody (1:500; Abcam). We counterstained the slices using 4′, 6′-diamidino-2-phenylindole (DAPI). Fluorescence images were acquired with a laser confocal microscope (FV500, Olympus) and the associated Fluoview2000 software. The objective lenses used were 20 and 40×.

### Data Analysis

Data are shown as means ± SEM. Statistical analyses were performed with GraphPad Prism 6.0 or StatView 4.5.1. ANOVAs or *t*-tests were used for statistical comparisons between groups as described in the main text. *P* < 0.05 was considered statistically significant.

## Results

### Decreased Intrinsic Excitability in CA1 Pyramidal Neurons of MD F1 Mice

**FIGURE 1:**
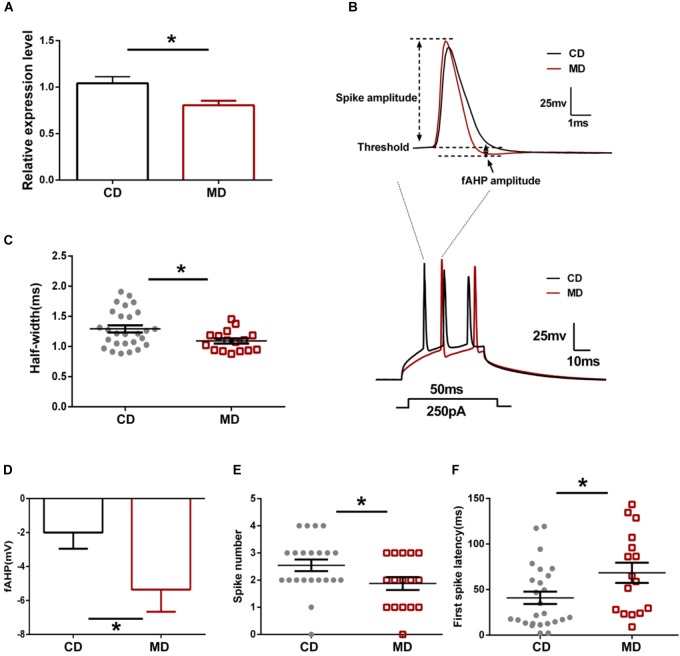
Different intrinsic excitability of CA1 pyramidal neurons in the MD F1 mice compared to the CD F1 mice. **(A)** qRT-PCR showing reduced expression of *Kcnmb2* in the hippocampus of MD F1 mice. Unpaired *t*-test, *n* = 5 mice for each group. **(B)** Sample recordings showing action potentials evoked by a depolarizing current injection. **Upper**: the first spike in response to a current injection (250 pA, 50 ms) into CA1 pyramidal neurons of CD F1 mice (black trace) and MD F1 mice (red trace). **Lower:** spikes trains evoked by a depolarizing current injection (250 pA, 50 ms) into CA1 pyramidal neurons of MD F1 (red trace) and CD F1 (black trace) mice. **(C)** Reduced action potential half-width in CA1 pyramidal neurons of MD F1 mice compared to CD F1 controls. Unpaired *t*-test, *n* = 27 cells from six CD F1 mice and *n* = 16 cells from five MD F1 mice. **(D)** Increased peak fAHP in CA1 pyramidal neurons of MD F1 mice. Unpaired *t*-test, *n* = 27 cells from six CD F1 mice and *n* = 17 cells from five MD F1 mice. **(E)** Reduced spike number in CA1 pyramidal neurons of MD F1 mice. Unpaired *t*-test, *n* = 22 cells from six CD F1 mice and *n* = 16 cells from five MD F1 mice. In **(C–E)**, depolarizing current was 250 pA and 50 ms in duration. **(F)** Prolonged latency to first spike in CA1 pyramidal neurons of MD F1 mice. Depolarizing current was 150 pA and 600 ms in duration. Unpaired *t-*test, *n* = 26 cells from six CD F1 mice and *n* = 16 cells from five MD F1 mice. ^∗^*P* < 0.05 means significant difference. All data are shown as means ± SEM.

Our recent study revealed decreased *Kcnmb2* mRNA expression associated with increased *Kcnmb2* promoter methylation in the hippocampus of MD F1 mice (Ryan et al., 2017). Changes in *Kcnmb2* expression could drive the neurophysiological and cognitive alterations observed in MD F1 animals (Ryan et al., 2017). Here, we initially reassessed *Kcnmb2* expression in offspring mice by qPCR and confirmed the reduced expression in the hippocampus of MD F1 animals relative to CD F1 mice (Figure 1A; unpaired *t-*test, *t* = 2.76, *P* < 0.05).

Since *Kcnmb2* encodes the BK channel β2 subunit mediating rapid inactivation of BK currents, we next assessed whether altered *Kcnmb2* expression affects intrinsic excitability of CA1 pyramidal neurons in MD F1 offspring mice. The passive membrane and firing properties of CA1 pyramidal neurons, including resting membrane potential (RP), input resistance, action potential (AP) threshold, AP half-width, AP numbers triggered by current injection and fAHP, were compared between MD F1 and CD F1 offspring mice. We found that the AP half-width of CA1 pyramidal neurons was smaller in MD F1 mice compared to that of CD F1 animals (Figures 1B,C; unpaired *t-*test, *t* = 2.37, *P* < 0.05). Moreover, we found that the fAHP amplitude measured in CA1 neurons, following either single spike or first spike in the context of burst firing triggered by current injection, was larger in MD F1 mice than in CD F1 controls (Figures 1B,D; unpaired *t-*test, *t* = 2.13, *P* < 0.05). When a depolarizing current was applied, CA1 neurons of MD F1 mice showed reduced spike numbers (Figures 1B,E; for a 250 pA current with 50 ms duration, unpaired *t*-test, *t* = 2.06, *P* < 0.05) and prolonged first spike latency (Figures 1B,F; for a 150 pA current with 600 ms duration, unpaired *t-*test, *t* = 2.23, *P* < 0.05) compared to CD F1 controls. However, there were no significant differences in RP, input resistance, spike amplitude and the AP threshold of CA1 pyramidal neurons between the two groups (Table 1; unpaired *t-*test, *P* > 0.05). We also compared the minimum current step to produce an action potential in CA1 pyramidal neurons and found no difference between CD F1 and MD F1 mice (Supplementary Figures S1A,B; Unpaired *t-*test, *P* > 0.05). In addition, we measured the action potential accommodation by a strong current step depolarization (600 ms, 500 pA). Both CD F1 and MD F1 CA1 pyramidal neurons showed obvious AP accommodation [Supplementary Figure 1C; Two-way Repeated Measure ANOVA with the between-subjects factor paternal diet and within-subjects factor inter-spike interval (ISI), paternal diet *F*_(1,33)_ = 0.51, *P* > 0.05; ISI *F*_(3,99)_ = 19.24, *P* < 0.0001; paternal diet × ISI *F*_(3,99)_ = 0.71, *P* > 0.05; Tukey’s multiple comparisons test, CD F1 *P* < 0.001 for 1^st^ and 3^nd^ ISI, and *P* < 0.0001 for 1^st^ and 4^th^ ISI; MD F1 *P* < 0.01 for 1^st^ and 4^th^ ISI]. We found only slight (not significant) increase in the 1^st^ ISI of MD F1 neurons compared to that of CD F1 neurons (Supplementary Figure S1C; Sidak’s multiple comparisons test, MD F1 vs. CD F1, *P* > 0.05). In addition, we found reduced number of APs elicited by a series of depolarizing current (50–525 pA, 600 ms in duration) injections in MD F1 neurons compared to CD F1 neurons (Supplementary Figure S1D; Two-way Repeated Measure ANOVA with the between-subjects factor paternal diet and within-subjects factor current injection, paternal diet *F*_(1,35)_ = 6.40, *P* < 0.05; current injection *F*_(19,665)_ = 266.2, *P* < 0.0001; paternal diet × current injection *F*_(19,665)_ = 2.32, *P* < 0.01; Sidak’s multiple comparisons test, MD F1 vs. CD F1, *P* < 0.05 at 225 or 250 pA current injection). In sum, CA1 neurons of MD F1 mice displayed reduced AP half-width, larger fAHP, an increased first spike latency and decreased firing numbers, which together point toward reduced intrinsic excitability of CA1 pyramidal neurons in MD F1 animals.

**Table 1:** Comparison of the basic membrane properties between CA1 pyramidal neurons of CD and MD F1 mice.

	RP (mV)	Threshold (mV)	AP amplitude (mV)	Input resistance (MΩ)
CD F1 (*n* = 27 cells, 6 mice)	-68.70 ± 0.6629	-43.54 ± 1.048	72.45 ± 2.104	345 ± 24.31
MD F1 (*n* = 17 cells, 5 mice)	-67.89 ± 1.728	-41.78 ± 2.413	76.58 ± 2.424	330 ± 24.15


### Altered Synaptic Excitation/Inhibition Ratio in CA1 Pyramidal Neurons of MD F1 Mice

**FIGURE 2:**
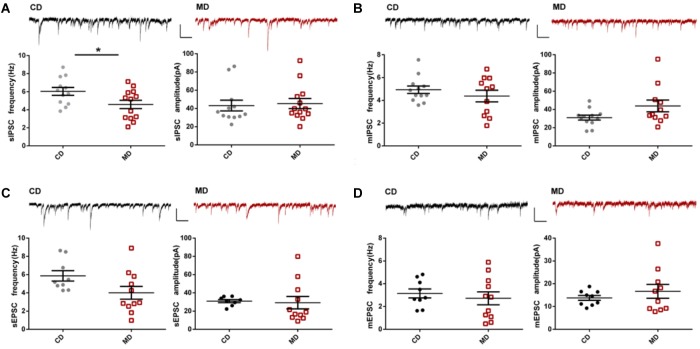
Altered synaptic transmission onto CA1 pyramidal neurons in MD F1 mice compared to CD F1 mice. **(A)** sIPSCs. **Upper:** sample recordings of sIPSCs in CD F1 (left, black) and MD F1 (right, red) mice. **Lower:** reduced sIPSC frequencies (left) and normal sIPSC amplitudes (right) in CA1 pyramidal neurons of MD F1 mice. Unpaired *t*-test, *P* < 0.05 for frequency, *P* > 0.05 for amplitude, *n* = 12 cells from five CD F1 mice and *n* = 13 cells from five MD F1 mice. The frequencies and amplitudes of mIPSCs **(B)**, sEPSCs **(C),** and mEPSCs **(D)** were not different between MD and CD F1 mice. Insets, sample traces recorded in CA1 pyramidal neurons of MD (red) and CD (black) F1 mice. In **(B)**, *n* = 12 cells from five CD F1 mice and *n* = 11 cells from five MD F1 mice. In **(C,D)**, *n* = 9 cells from five CD F1 mice and *n* = 11 cells from five MD F1 mice. Scale bars: 100 pA, 250 ms. ^∗^*P* < 0.05 means significant difference. All data are shown as means ± SEM.

Next, we investigated excitatory and inhibitory synaptic transmission in MD F1 and CD F1 mice by recording spontaneous and miniature post-synaptic currents (PSCs) in CA1 pyramidal neurons of these mice. We found no significant changes in both sEPSCs and mEPSCs, indicating overall excitatory synaptic transmission onto CA1 pyramidal neurons was unaltered in MD F1 mice (Figures 2C,D; unpaired *t-*test, *P* > 0.05 compared to the CD group). However, IPSC recordings revealed decreased sIPSC frequency, and unaltered sIPSC amplitude in CA1 pyramidal neurons of MD F1 offspring relative to CD F1 controls (Figure 2A; unpaired *t-*test, *t* = 2.28 and *P* < 0.05 for sIPSC frequency; *t* = 0.27 and *P* > 0.05 for sIPSC amplitude). This suggests an attenuation in spontaneous inhibitory synaptic activity onto CA1 pyramidal neurons of MD F1 mice, caused by either reduced presynaptic GABA release and/or reduced numbers of inhibitory synapses. We also measured mIPSCs in the presence of tetrodotoxin (TTX), an action potential blocker. Neither mIPSC amplitude nor mIPSC frequency of CA1 pyramidal neurons was changed in MD F1 offspring in comparison to CD F1 controls (Figure 2B; unpaired *t-*test, *P* > 0.05). To determine whether there was an alteration in the number of release sites between GABAergic interneurons and CA1 pyramidal neurons in the MD F1 mice compared to the CD F1 mice, we analyzed the multiplicity index for both GABA and glutamate release following the method described in a previous study (Groc et al., 2003). We found that neither the average number of GABA release sites or glutamate release sites changed in the hippocampus of MD F1 mice compared to CD F1 mice (Supplementary Figures S2A,B; unpaired *t-*test, *P* > 0.05). Therefore, other mechanisms rather than reduced number of GABA release sites or connectivity between GABAergic interneurons and CA1 pyramidal neurons should contribute to reduced sIPSC frequency observed in MD F1 neurons. Our results thus identified a selective effect on sIPSCs but not mIPSCs of CA1 pyramidal neurons in MD F1 offspring.

### Overexpression of *Kcnmb2* in Dorsal Hippocampus Masked MD-F1-Associated Alterations in Intrinsic Excitability of CA1 Pyramidal Neurons

**FIGURE 3:**
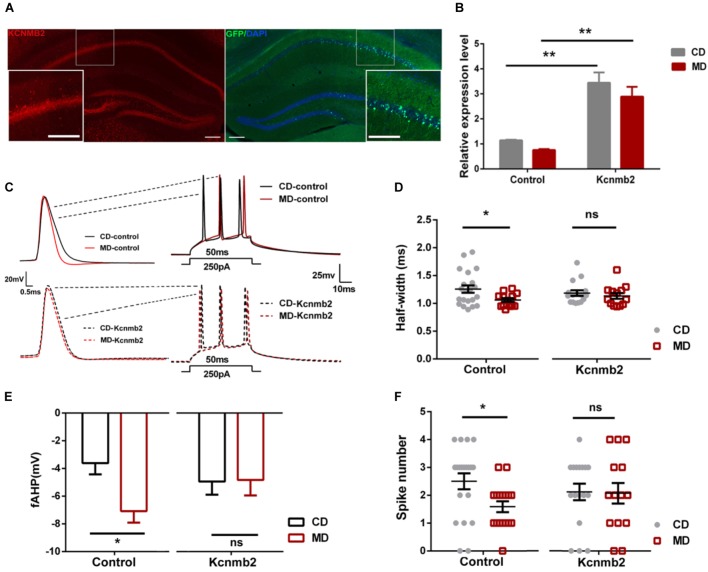
Overexpression of *Kcnmb2* in the hippocampus masked MD-F1-associated alterations in intrinsic excitability of CA1 pyramidal neurons **(A)** Representative images showing AAV-associated GFP (right) and Kcnmb2 expression (left) in dorsal hippocampus. Red, green, and blue fluorescence reflects Kcnmb2-, GFP-, and DAPI-associated signal, respectively. Scale bars represent 100 μm. The insets are higher magnification images of the boxed area. **(B)** Quantification of the relative *Kcnmb2* mRNA expression in the hippocampus after delivery of AAV-*Kcnmb2* or AAV-control virus into CA1 region of MD and CD F1 mice. Two-way ANOVA followed by Tukey’s multiple comparisons test, *n* = 4, 4, 6, and 6 samples for CD-control, MD-control, CD-*Kcnmb2*, and MD-*Kcnmb2* groups, respectively. **(C)** Sample recordings in virus-infected CA1 pyramidal neurons (GFP^+^) showing action potentials evoked by a depolarizing current injection in CD and MD F1 mice. **Left**: the first spike in response to a current injection (250 pA, 50 ms) into CA1 pyramidal neuron. **Right**: spikes trains evoked by a depolarizing current injection (250 pA, 50 ms) into CA1 pyramidal neurons. CD F1 neuron (black) and MD F1 neuron (red) infected by AAV-control (solid traces) or AAV-*Kcnmb2* (dashed traces) virus. Comparisons of action potential half-width **(D)**, fAHP **(E)**, and spike numbers **(F)** between CD and MD F1 neurons infected by control or *Kcnmb2* virus. Two-way ANOVA followed by Sidak’s multiple comparisons test, 5–6 mice per group. AP half-width: *n* = 21 cells for CD-control, *n* = 13 cells for MD-control, *n* = 16 cells for CD-*Kcnmb2* and *n* = 13 cells for MD-*Kcnmb2*; fAHP: *n* = 14 cells for CD-control, *n* = 15 cells for MD-control, *n* = 16 cells for CD-*Kcnmb2* and *n* = 14 cells for MD-*Kcnmb2*; Spike number: *n* = 20 cells for CD-control, *n* = 17 cells for MD-control, *n* = 17 cells for CD-*Kcnmb2* and *n* = 14 cells for MD-*Kcnmb2*. ^∗^*P* < 0.05, ^∗∗^*P* < 0.01, and ^∗∗∗^*P* < 0.01 means significant difference, ns means not significant. All data are shown as means ± SEM.

We found reduced *Kcnmb2* expression in the hippocampus of MD F1 offspring, which was accompanied by reduced intrinsic excitability and inhibitory synaptic activity in CA1 pyramidal neurons. To examine whether a causal relationship exists between altered *Kcnmb2* expressions on the one hand and changes in excitability/inhibitory synaptic activity on the other hand, we investigated whether overexpression of *Kcnmb2* in CA1 dispels MD F1-related changes in neuronal excitability and synaptic transmission. Toward this end, AAV engineered to overexpress *Kcnmb2* (AAV-*Kcnmb2*) or GFP (AAV-control) was injected into the CA1 region of the dorsal hippocampus prior to electrophysiological analyses. First, we confirmed successful AAV transduction in hippocampal area CA1 based on GFP-associated fluorescence, as well as Kcnmb2 expression measured by immunofluorescence staining (Figure 3A). Next, *Kcnmb2* expression in the hippocampus was quantified by qPCR: AAV-*Kcnmb2* increased *Kcnmb2* expression in the hippocampus of both CD and MD F1 mice compared to AAV-control virus (Figure 3B; Two-way ANOVA with the between-subjects factors AAV treatment and paternal diet, AAV treatment *F*_(1,16)_ = 38.43, *P* < 0.0001; paternal diet *F*_(1,16)_ = 0.05, *P* > 0.05; paternal diet × AAV treatment *F*_(1,16)_ = 1.74, *P* > 0.05; Tukey’s multiple comparisons test, *P* < 0.01 for CD-*Kcnmb2* vs. CD-control and *P* < 0.01 for MD-*Kcnmb2* vs. MD-control). There was no difference between CD-*Kcnmb2* and MD-*Kcnmb2* mice (*P* > 0.05).

Our whole-cell current-clamp recordings revealed that the MD F1 neurons infected by control virus (MD-control) displayed reduced AP half-width (Figures 3C,D; Two-way ANOVA with the between-subjects factors paternal diet and AAV treatment, paternal diet *F*_(1,59)_ = 4.48, *P* < 0.05; AAV treatment *F*_(1,59)_ = 0.0001, *P >* 0.05; Paternal diet × AAV treatment *F*_(1,59)_ = 1.57, *P* > 0.05), larger fAHP (Figures 3C,E; Two-way ANOVA with the between-subjects factors paternal diet and AAV treatment, paternal diet *F*_(1,55)_ = 4.11, *P <* 0.05; AAV treatment *F*_(1,55)_ = 0.29, *P >* 0.05; paternal diet × AAV treatment *F*_(1,55)_ = 3.65, *P* = 0.06) and decreased firing numbers (Figures 3C,F; Two-way ANOVA with the between-subjects factors paternal diet and AAV treatment, paternal diet factor *F*_(1,64)_ = 3.74, *P* = 0.07; AAV treatment *F*_(1,64)_ = 0.03, *P >* 0.05; paternal diet × AAV treatment *F*_(1,64)_ = 2.23, *P* > 0.05) compared to control-virus-infected CD F1 neurons (CD-control, Sidak’s multiple comparisons test, *P* < 0.05), consistent with the findings in non-infected CA1 pyramidal neurons of MD F1 mice described above. In contrast, MD F1 pyramidal neurons infected by AAV-*Kcnmb2* (MD-*Kcnmb2*) displayed similar firing properties as infected CD F1 neurons (CD-*Kcnmb2*), including AP half-width, fAHP and firing numbers (Figures 3C–F; Sidak’s multiple comparisons test, *P* > 0.05). We also compared AP threshold and first spike latency between CD F1 and MD F1 CA1 neurons infected by control or *Kcnmb2* virus. We found that, AP threshold were similar among four groups (Supplementary Figure S3A; Two-way ANOVA with the between-subjects factors paternal diet and AAV treatment, paternal diet *F*_(1,56)_ = 0.07, *P* > 0.05; AAV treatment *F*_(1,56)_ = 0.29, *P >* 0.05; Paternal diet × AAV treatment *F*_(1,56)_ = 1.08, *P* > 0.05). However, the MD-control neurons displayed slightly increased first spike latency compared to the CD-control neurons (Supplementary Figure S3B; Two-way ANOVA with the between-subjects factors paternal diet and AAV treatment, paternal diet *F*_(1,48)_ = 4.06, *P* < 0.05; AAV treatment *F*_(1,48)_ = 0.23, *P >* 0.05; Paternal diet × AAV treatment *F*_(1,48)_ = 0.80, *P* > 0.05; Sidak’s multiple comparisons test, *P* = 0.06), while MD-*Kcnmb2* neurons displayed identical first spike latency to CD-*Kcnmb2* neurons (Supplementary Figure S3B; Sidak’s multiple comparisons test, *P* > 0.05). Therefore our results indicated that overexpression of *Kcnmb2* masked alterations in intrinsic excitability of offspring CA1 pyramidal neurons caused by a paternal methyl-donor supplementation.

**FIGURE 4:**
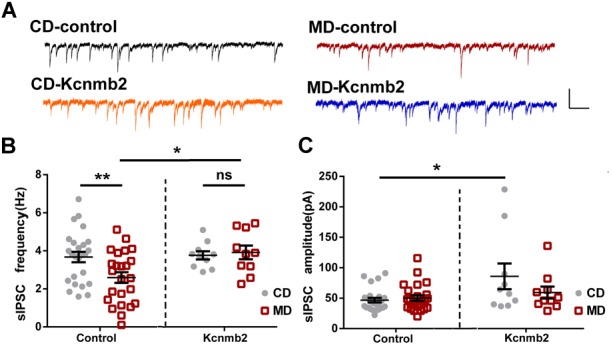
Overexpression of *Kcnmb2* in CA1 region of dorsal hippocampus rescued alterations in synaptic transmission onto CA1 pyramidal neurons in MD F1 mice. **(A)** Sample sIPSCs recorded from virus-infected CA1 pyramidal neurons (GFP^+^) in MD and CD F1 mice. Comparisons of sIPSC frequencies **(B)** and sIPSC amplitudes **(C)** between CD and MD F1 neurons infected by control or *Kcnmb2* virus. Two-way ANOVA followed by Tukey’s multiple comparisons test, CD-control *n* = 25, MD-control *n* = 24, CD-*Kcnmb2 n* = 10, and MD-*Kcnmb2 n* = 10 neurons from six mice per group. ^∗^*P* < 0.05, ^∗∗^*P* < 0.01 means significant difference. All data are shown as means ± SEM.

Similar to what we found in non-infected CA1 pyramidal neurons of MD F1 mice, neurons infected by control GFP virus in MD F1 mice (MD-control) showed reduced sIPSC frequency compared to CD-control neurons (Figures 4A,B; Two-way ANOVA with the between-subjects factors AAV treatment and paternal diet, AAV treatment *F*_(1,65)_ = 4.39, *P* < 0.05; Paternal diet *F*_(1,65)_ = 1.90, *P* > 0.05; Paternal diet × AAV treatment *F*_(1,65)_ = 3.360, *P* = 0.07; Tukey’s multiple comparisons test, *P* < 0.01 for MD vs. CD F1 neurons infected by control virus). Importantly, we found that overexpression of *Kcnmb2* in CA1 pyramidal neurons abolished such alterations in inhibitory synaptic transmission as observed in MD F1 offspring mice. Specifically, the MD-*Kcnmb2* pyramidal neurons displayed similar sIPSC frequencies as the CD-*Kcnmb2* neurons (Figures 4A,B; Tukey’s multiple comparisons test, *P* > 0.05 for MD vs. CD F1 neurons infected by *Kcnmb2*-AAV). Moreover, CA1 pyramidal neurons infected by *Kcnmb2* virus showed increased sIPSC frequencies compared to those infected by control virus in MD F1 mice (Figures 4A,B, Tukey’s multiple comparisons test, *P* < 0.05 for MD-*Kcnmb2* vs. MD-control neurons). As expected, overexpression of *Kcnmb2* or GFP did not cause differences in sIPSC amplitudes between infected MD and CD F1 neurons (Figures 4A,C; two-way ANOVA with the between-subjects factors AAV treatment and paternal diet, AAV treatment *F*_(1,65)_ = 7.82, *P* < 0.01; Paternal diet *F*_(1,65)_ = 1.81, *P* > 0.05; Paternal diet × AAV treatment *F*_(1,65)_ = 3.04, *P* = 0.08; Tukey’s multiple comparisons test, *P* > 0.05 for MD vs. CD F1 neurons). However, CA1 pyramidal neurons infected by *Kcnmb2* virus did show increased sIPSC amplitudes in CD F1 mice, but not in MD F1 mice (Figures 4A,C; Tukey’s multiple comparisons test, *P* < 0.05 for CD F1 mice receiving *Kcnmb2* vs. control virus). We did not observed significant difference in sEPSC, mEPSC or mIPSC among four groups (data not shown). Therefore, our results confirmed that overexpression of *Kcnmb2* abolished alterations in both intrinsic excitability and synaptic transmission in CA1 pyramidal neurons of MD F1 offspring.

### Overexpressions of *Kcnmb2* in Dorsal CA1 Rescued the LTP Deficits Observed in MD F1 Mice

**FIGURE 5:**
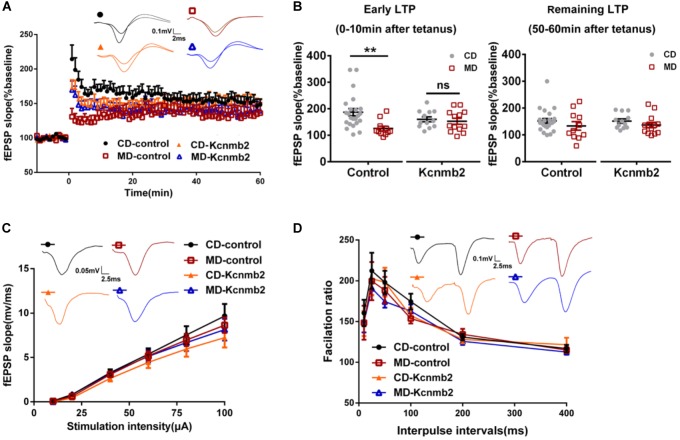
Overexpression of *Kcnmb2* in CA1 region of dorsal hippocampus rescued LTP deficits in MD F1 mice. **(A)** LTP induced with a 100 Hz, 1 s tetanus in four groups of mice. Inserts, sample fEPSP traces recorded at 10 min post-tetanus. Three-way ANOVA with the between-subjects factors paternal diet (MD vs. CD) and AAV treatment (AAV-Kcnmb2 vs. AAV-control), and the within-subjects factor time. *n* = 11 slices from six MD-control mice, *n* = 23 slices from eight CD-control mice; *n* = 13 slices from six MD-*Kcnmb2* mice and *n* = 12 slices from seven CD-*Kcnmb2* mice. **(B)** Comparisons of early LTP (left, 0–10 min post-tetanus) and remaining LTP (right, 50–60 min post-tetanus) in SC-CA1 synapses pathway between CD and MD F1 neurons infected by control or *Kcnmb2* virus. Two-way ANOVA followed by Tukey’s multiple comparisons test, *n* = 11 slices from six MD-control mice, *n* = 23 slices from eight CD-control mice; *n* = 13 slices from six MD-*Kcnmb2* mice and *n* = 12 slices from seven CD-*Kcnmb2* mice. The input–output curve **(C)** and paired-pulse ratio (PPR) **(D)** indicating no significant differences in basal synaptic transmission of SC-CA1 pathway between MD and CD F1 mice receiving control or *Kcnmb2* virus injection. Three-way ANOVA, *n* = 23 slices from six MD-control mice, *n* = 35 slices from eight CD-control mice, *n* = 25 slices from six MD-*Kcnmb2* mice and *n* = 28 slices from seven CD-*Kcnmb2* mice. Inserts in **(C)**, sample fEPSPs evoked by 40 μA (100 μs) stimulation delivered to SC-CA1 pathway. Inserts in **(D)**, sample fEPSPs evoked by paired-pulse stimulation (40 μA, 100 μs) with ISI of 50 ms. ^∗∗^*P* < 0.01 means significant difference. All data are shown as means ± SEM.

In a previous study, we reported abnormal LTP and memory impairments in MD F1 offspring mice (Ryan et al., 2017). In this study, we further tested whether AAV-mediated overexpression of *Kcnmb2* in CA1 of dorsal hippocampus rescued LTP deficits in MD F1 mice. LTP was measured at Schaffer Collateral/CA1 synapses in acute brain slices prepared from MD or CD F1 offspring receiving stereotaxic virus injection into the CA1 region of dorsal hippocampus. Three-way ANOVA with the between-subjects factors paternal diet (MD vs. CD) and AAV treatment (AAV-Kcnmb2 vs. AAV-control) and the within-subjects factor time after LTP induction revealed a significant interaction between paternal diet and time (Figure 5A; *F*_(1,59)_ = 1.94, *P* < 0.0001), as well as a significant interaction between the factors paternal diet, AAV treatment and time (Figure 5A, *F*_(1,59)_ = 3.15, *P* < 0.0001), indicating that the paternal MD diet was associated with altered temporal LTP profiles that were modified by AAV-Kcnmb2 treatment of MD F1 offspring. To our surprise, we did not find LTP abnormality in MD F1 mice comparing to CD F1 mice after either *Kcnmb2* or control virus injection (Figures 5A,B; fEPSPs measured at 50–60 min post-tetanus, Two-way ANOVA with the between-subjects factors paternal diet and AAV treatment, *P* > 0.05). Those results discrepancy may be due to difference in the age of animals, elder mice (about 8 month of age) in current study while much younger ones (about 3 month of age) in our previous study (Ryan et al., 2017). Nevertheless, our experiments did reveal reduced early LTP measured at 0–10 min post-tetanus in the SC-CA1 synapses of the MD F1 mice compared to the CD F1 mice that received same control virus injection (Figures 5A,B; Two-way ANOVA with the between-subjects factors paternal diet and AAV treatment, paternal diet *F*_(1,55)_ = 6.65, *P* < 0.05; paternal diet × AAV treatment *F*_(1,55)_ = 4.17, *P* < 0.05; Tukey’s multiple comparisons test, CD-control vs. MD-control, *P* < 0.01). In contrast, the early LTP in the SC-CA1 synapses of MD F1 mice receiving AAV-*Kcnmb2* injection (MD-*Kcnmb2*) was comparable to that recorded in both CD-*Kcnmb2* and CD-control mice (two-way ANOVA followed by Tukey’s multiple comparisons test, *P* > 0.05), indicating that virus-mediated overexpression of *Kcnmb2* abolished early LTP alteration in SC-CA1 synapses of MD F1 offspring, but had no significant effect on CD F1 mice. Basal synaptic transmission (Figure 5C; Three-way ANOVA with the between-subjects factors paternal diet and AAV treatment, as well as the within-subjects factor stimulation intensity, *F*_(1,5)_ = 0.38, *P >* 0.05) and PPR (Figure 5D; Three-way ANOVA with the between-subjects factors paternal diet and AAV treatment, as well as the within-subjects factor inter-stimulus interval, *F*_(1,5)_ = 0.57, *P* > 0.05) were undistinguishable among four groups.

## Discussion

Our previous study showed that excessive paternal methyl donor intake prior to mating results in *Kcnmb2* promoter hypermethylation associated with reduced *Kcnmb2* expression which may relate to LTP deficits, abnormalities in hippocampal theta oscillations as well as spatial learning impairments in MD F1 offspring mice (Ryan et al., 2017). Here, we analyzed CA1 neuronal properties in MD F1 mice and found decreased intrinsic excitability as well as reduced inhibitory synaptic transmission in these animals. AAV-mediated *Kcnmb2* overexpression in dorsal CA1 abolished these changes in neuronal excitability and synaptic transmission and improved LTP, as well as spatial learning and memory impairments in MD F1 mice [for behavioral results, see our previous study (Ryan et al., 2017)], supporting a model whereby repression of *Kcnmb2* drives neural and cognitive alterations that occur in MD F1 offspring as a consequence of excessive paternal methyl donor intake.

*Kcnmb2* is expressed mainly in the brain and encodes the β2 auxiliary subunit of BK channels, which confers fast inactivation of the channels (Wallner et al., 1999; Xia et al., 2003; Sun et al., 2012). The β2-containing BK channels have been identified in hippocampus, neocortex, and lateral amygdala pyramidal neurons (Sailer et al., 2006; Sausbier et al., 2006; Contet et al., 2016). More recent research showed that β2 subunit activity can regulate suprachiasmatic nucleus rhythm and circadian behavior by inactivation of BK currents (Whitt et al., 2016). Also, a SNP (RS9637454) of *Kcnmb2* was reported to be strongly associated with hippocampal sclerosis, a comorbid neuropathological feature of AD (Gibson et al., 2014; Katsumata et al., 2017).

BK channels, ubiquitously distributed in a variety of neuronal and non-neuronal tissues, play a role in dampening excitatory signals via repolarization of the membrane potential and limiting Ca^2+^ entry through voltage-dependent Ca^2+^ channels. Therefore, BK channels represent negative feedback regulators of membrane excitability and cytoplasmic Ca^2+^ concentration (Lee and Cui, 2010; Rothberg, 2012; Contreras et al., 2013). By mediating fAHP, BK channels exert a powerful control on action potential duration and neuronal firing ability, regulating neurotransmitter release and dendritic excitability (Bock and Stuart, 2016). Both loss and gain of BK channel function have been associated with neurological and psychiatric disorders, such as epilepsy, schizophrenia, autism, mental retardation, and chronic pain (Griguoli et al., 2016; Wang et al., 2016). In hippocampal pyramidal cells, BK channels are present in the presynaptic membrane facing the synaptic cleft, as well as in the head of dendritic spines, in close proximity to the post-synaptic specialization of glutamatergic synapses (Hu et al., 2001; Sailer et al., 2006).

In a previous study, we found that paternal exposure to a methyl donor-rich diet inhibited *Kcnmb2* expression and increased *Kcnmb2* promoter methylation in F1 offspring animals. We predicted that, as a consequence, the activity of BK channels in MD F1 offspring mice would be facilitated, leading to suppression of neuronal excitability. Indeed, we found not only increased fAHP and reduced half-width of action potentials, but also increased first spike latency and decreased firing numbers in CA1 pyramidal neurons of MD F1 mice. Increased fAHP and reduced half-width of action potentials could affect Ca^2+^ entry during repolarization which then influences neurotransmitter release, immediate intracellular signal transduction, as well as longer-term Ca^2+^-mediated changes in neurons, for instance gene transcription, kinase activation, and synaptic plasticity (Matthews et al., 2008). Supportively, we did observe reduced inhibitory synaptic transmission and early LTP deficit in MD F1 mice. Moreover, *Kcnmb2* overexpression abolished those changes in fAHP and AP half-width, meanwhile rescued abnormalities in synaptic transmission and LTP, suggesting that intrinsic excitability changes should be primary. It is also possible that reduced inhibitory synaptic activity is just a network compensation for reduced excitability in the pyramidal neurons of MD F1 mice. Such compensation may partially explain why we only observed early LTP deficit in MD F1 mice.

On the other hand, since fAHP depends on BK channel activity, overexpression of *Kcnmb2* leading to inhibition of BK channels activity should produce an increase in fAHP. However, as shown in Figure 3E, we only observed an increasing trend, but not a significant increase of fAHP in MD F1 pyramidal neurons overexpressing *Kcnmb2*. In addition, given the difference in the relative expression of *Kcnmb2*, we would expect that there is a significant difference in the half-width between CD-control and CD-Kcnmb2, also between MD-control and MD-Kcnmb2. However, we only observed significant differences between CD- and MD-control, not the other pairs. Those unexpected results seem to be hard to explain. Nevertheless, there are four types of β subunits encoded by the *Kcnmb1–4* genes respectively, which modify the gating properties of the BK channels. Both β2 and β3 subunits are expressed in neuron while β4 is expressed within the brain. Overexpression of one subunit, for example Kcnmb2, may cause down-regulation of other subunits therefore compensate or mask the effect of *Knmb2* overexpression on neuronal excitability. Also, in our study, relative *Kcnmb2* expression was quantified with qPCR analysis which may not intuitively reflect the level of protein expression. Moreover, in our study, *Kcnmb2* overexpression was mediated by viral infection. Since it is very difficult to precisely control the infection rate, sampling variation may be relatively high.

Besides alterations in neuronal excitability, we also found reduced sIPSC frequencies but unchanged sIPSC amplitudes in CA1 pyramidal neurons of MD F1 mice. Neither mIPSC amplitudes nor mIPSC frequencies of CA1 pyramidal neurons were altered in the presence of TTX (to block firing), indicating that the release probability or quantal content of GABA may not change. Moreover, our data suggested that the average number of GABA release sites in CA1 pyramidal neurons was not changed in MD F1 mice. Therefore, it is possible that the attenuation in inhibitory synaptic activity onto CA1 pyramidal neurons of MD F1 mice is due to reduced GABA release triggered by hyperactivity of presynaptic BK channels and thus lower excitability of presynaptic GABAergic neurons. Nevertheless, more experiments, such as testing the evoked IPSC, the paired pulses ratio or the coefficient of variation in IPSC, should be done to confirm whether the release probability of GABA is changed or not. Previous studies demonstrated that presynaptic BK channels inhibit both glutamate and GABA release (Wang, 2008; Martire et al., 2010; Samengo et al., 2014). However, we did not find changes in the excitatory synaptic transmission or probability of glutamate release at SC-CA1 synapses of MD F1 mice. It is worth to be mentioned, our fEPSP recordings was done without a GABA-A receptor antagonist and we did not found difference among four groups. However, since decrease in the inhibitory synaptic transmission while no change in the excitatory synaptic transmission was observed in MD F1 mice, it will be interesting to test whether fEPSP among four groups are still same after blocking inhibitory transmission with a GABA-A receptor antagonist. Altogether, our data suggested decreased inhibitory and unchanged excitatory synaptic transmission in CA1 microcircuit of MD F1 mice. The physiological significance of such alterations in synaptic transmission is uncertain. One possibility is a compensatory adaptation to the reduced neuronal or dendritic excitability in MD F1 mice due to BK channel hyperactivity. Supportively, we found an important difference in the post-tetanic potentiation rather than LTP of MD F1 mice, which is NMDA receptor-independent, but directly related to the degree of depolarization, opening of calcium channels and residual calcium in the synaptic end. In general, BK channels help to maintain a physiological range of circuit output, so that both insufficient and excessive BK channel activities can have detrimental effects on brain function.

A growing number of studies document the role BK channels play in the regulation of learning and memory. For example, it was reported that BK channel knockout mice or mice with deficient function of BK channels require more time to learn the Morris water maze (Oh et al., 2003; Typlt et al., 2013) and that intracranial injections of BK blockers dampened the acquisition of an eye blink conditioning task (Matthews et al., 2008; Matthews and Disterhoft, 2009). A previous study reported that chronic BK channel activation can improve memory deficits in a mouse model of Alzheimer’s disease (Ye et al., 2010). One the other hand, it was shown that increased activity of presynaptic BK channels accounts for reduced excitatory transmission in the hippocampus of an AD mouse model and thus treatments enhancing BK channel activity can aggravate synaptic dysfunction (Hu et al., 2001; Ye et al., 2010). Additional findings suggested that both decreased and increased BK channel activity may lead to mental retardation (Contet et al., 2016). Our study provided further evidence that increased BK channel activity by *Kcnmb2* down-regulation inhibits neuronal excitability and presynaptic GABA release, impairs synaptic plasticity in the hippocampal network and leads to spatial memory deficit. Meanwhile, we found that, although *Kcnmb2* overexpression and resulting BK channel inactivation rescued hippocampal dysfunction caused by *Kcnmb2*-deficit and BK channel hyperactivity, the same treatment led to synaptic inhibition (increased sIPSCs amplitude) and slight reduction (not significant yet) of early LTP in CD F1 mice with otherwise normal *Kcnmb2* expression and BK channel activity. Therefore our findings support the concept that both insufficient and excessive BK channel activities could be detrimental to brain function.

A growing body of evidence supports the notion that pre- and post-natal nutrition is critical for healthy neurological development. Nutrients can modulate epigenetic marks in the genome as well as gene expression patterns thus resulting in long-term phenotypic changes (Zovkic et al., 2013; Barker et al., 2017; Park et al., 2017). Our results indicate that altered BK channel activity induced by paternal nutrients can disrupt circuit function in offspring hippocampus and lead to impairment in learning and memory. Therefore, subpopulations of BK channels or its auxiliary subunits could be a potential therapeutic target to correct diverse pathologies and neurological dysfunctions associated with CNS-wide loss or gain of function of BK channels.

## Author Contributions

MY, LG, NL, KH, HG, and SL performed experiments. WS, XR and YL contributed to data analyses. DE and YZ supervised the experiments and drafted the manuscript. All authors read and approved the final manuscript.

## Conflict of Interest Statement

The authors declare that the research was conducted in the absence of any commercial or financial relationships that could be construed as a potential conflict of interest.

## Supplementary Material

FIGURE S1 Comparisons of minimum current to induce an AP and AP adaptation between CA1 pyramidal neurons of the MD and CD F1 mice. **(A)** Minimum current to induce an AP with a series of step current injections (50 ms in duration). Unpaired *t*-test, *n* = 25 cells from six CD F1 mice and *n* = 19 cells from five MD F1 mice. **(B)** Minimum current to induce an AP with a series of step current injections (600 ms in duration). Unpaired *t*-test, *n* = 30 cells from six CD F1 mice and *n* = 18 cells from five MD F1 mice. **(C)** Inter-spike intervals (ISIs) of CA1 pyramidal neurons in CD F1 mice and MD F1 mice. Depolarization current applied to induce a train of spikes was 600 ms and 500 pA. Two-way ANOVA followed by Sidak’s multiple comparisons test (CD F1 vs. MD F1 mice) or Tukey’s multiple comparisons test (2^nd^, 3^rd^, or 4^th^ ISI vs. 1^st^ ISI), *n* = 23 cells from six CD F1 mice and *n* = 12 cells from five MD F1 mice. **(D)** Action potentials in response to increasing current injections (600 ms duration, stepping from 50 to 525 pA in 25 pA increments). Two-way ANOVA followed by Sidak’s (CD F1 vs. MD F1 mice) or Tukey’s (different current injection) multiple comparisons test, *n* = 20 cells from six CD F1 mice and *n* = 17 cells from five MD F1 mice. ^∗^*P* < 0.05, ^∗∗^*P* < 0.01, ^∗∗∗^*P* < 0.001, or ^∗∗∗∗^*P* < 0.0001 means significant difference. All data are shown as means ± SEM.

FIGURE S2 Comparisons of GABA and glutamate release site connectivity with CA1 pyramidal neurons in the MD and CD F1 mice. **(A)** GABA multiplicity factor. Unpaired *t*-test, *n* = 9 cells from four CD F1 mice and *n* = 8 from four MD F1 mice. **(B)** Glutamate multiplicity factor. Unpaired *t*-test, *n* = 8 cells from four CD F1 mice and *n* = 11 from four MD F1 mice. The multiplicity factor was calculated according to previous description (Groc et al., 2003). All data are shown as means ± SEM.

FIGURE S3 Comparisons of AP threshold and first spike latency between CD F1 and MD F1 CA1 neurons infected by control or *Kcnmb2* virus. **(A)** AP threshold were similar among four groups. Two-way ANOVA followed by Sidak’s multiple comparisons test, *n* = 15 cells for CD-control, *n* = 15 cells for MD-control, *n* = 16 cells for CD-*Kcnmb2*, and *n* = 14 cells for MD-*Kcnmb2*. **(B)** First spike latency. Two-way ANOVA followed by Sidak’s multiple comparisons test, *n* = 14 cells for CD-control, *n* = 14 cells for MD-control, *n* = 13 cells for CD-*Kcnmb2*, and *n* = 11 cells for MD-*Kcnmb2*. All data are shown as means ± SEM.
