# Supplementary figures and images for: Overexpression of Kcnmb2 in Dorsal CA1 of Offspring Mice Rescues Hippocampal Dysfunction Caused by a Methyl Donor-Rich Paternal Diet
Source: Front Cell Neurosci. 2018 Oct 23;12:360. doi: 10.3389/fncel.2018.00360 (PMC6206260; doi:10.3389/fncel.2018.00360)

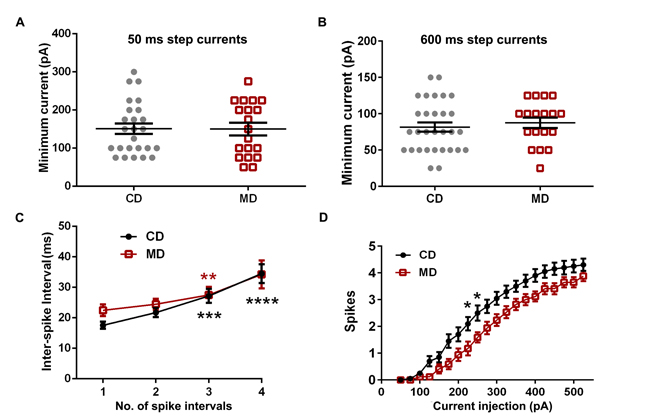

Supplement: FIGURE S1 — Comparisons of minimum current to induce an AP and AP adaptation between CA1 pyramidal neurons of the MD and CD F1 mice. (A) Minimum current to induce an AP with a series of step current injections (50 ms in duration). Unpaired t-test, n = 25 cells from six CD F1 mice and n = 19 cells from five MD F1 mice. (B) Minimum current to induce an AP with a series of step current injections (600 ms in duration). Unpaired t-test, n = 30 cells from six CD F1 mice and n = 18 cells from five MD F1 mice. (C) Inter-spike intervals (ISIs) of CA1 pyramidal neurons in CD F1 mice and MD F1 mice. Depolarization current applied to induce a train of spikes was 600 ms and 500 pA. Two-way ANOVA followed by Sidak’s multiple comparisons test (CD F1 vs. MD F1 mice) or Tukey’s multiple comparisons test (2nd, 3rd, or 4th ISI vs. 1st ISI), n = 23 cells from six CD F1 mice and n = 12 cells from five MD F1 mice. (D) Action potentials in response to increasing current injections (600 ms duration, stepping from 50 to 525 pA in 25 pA increments). Two-way ANOVA followed by Sidak’s (CD F1 vs. MD F1 mice) or Tukey’s (different current injection) multiple comparisons test, n = 20 cells from six CD F1 mice and n = 17 cells from five MD F1 mice. ∗P < 0.05, ∗∗P < 0.01, ∗∗∗P < 0.001, or ∗∗∗∗P < 0.0001 means significant difference. All data are shown as means ± SEM. [file Image_1.JPEG]

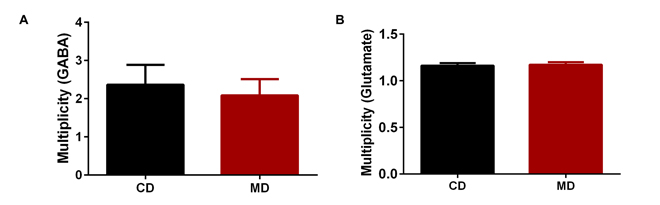

Supplement: FIGURE S2 — Comparisons of GABA and glutamate release site connectivity with CA1 pyramidal neurons in the MD and CD F1 mice. (A) GABA multiplicity factor. Unpaired t-test, n = 9 cells from four CD F1 mice and n = 8 from four MD F1 mice. (B) Glutamate multiplicity factor. Unpaired t-test, n = 8 cells from four CD F1 mice and n = 11 from four MD F1 mice. The multiplicity factor was calculated according to previous description (Groc et al., 2003). All data are shown as means ± SEM. [file Image_2.JPEG]

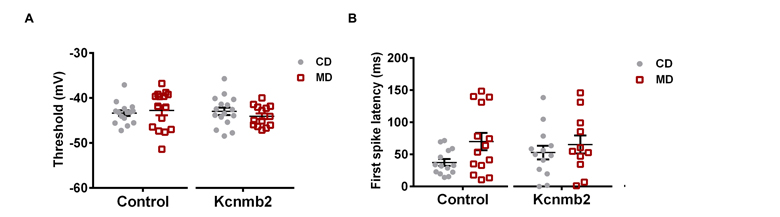

Supplement: FIGURE S3 — Comparisons of AP threshold and first spike latency between CD F1 and MD F1 CA1 neurons infected by control or Kcnmb2 virus. (A) AP threshold were similar among four groups. Two-way ANOVA followed by Sidak’s multiple comparisons test, n = 15 cells for CD-control, n = 15 cells for MD-control, n = 16 cells for CD-Kcnmb2, and n = 14 cells for MD-Kcnmb2. (B) First spike latency. Two-way ANOVA followed by Sidak’s multiple comparisons test, n = 14 cells for CD-control, n = 14 cells for MD-control, n = 13 cells for CD-Kcnmb2, and n = 11 cells for MD-Kcnmb2. All data are shown as means ± SEM. [file Image_3.JPEG]
